# A Low Profile Ultra-Wideband Antenna with Reconfigurable Notch Band Characteristics for Smart Electronic Systems

**DOI:** 10.3390/mi13111803

**Published:** 2022-10-22

**Authors:** Abir Zaidi, Wahaj Abbas Awan, Adnan Ghaffar, Mohammed S. Alzaidi, Mohammad Alsharef, Dalia H. Elkamchouchi, Sherif S. M. Ghoneim, Turki E. A. Alharbi

**Affiliations:** 1 Laboratory EEA & TI, Faculty of Science and Techniques (FSTM) Mohammedia, Hassan II University, Casablanca 20000, Morocco; 2 Department of Information and Communication Engineering, Chungbuk National University, Chengju 28644, Korea; 3 Department of Electrical and Electronic Engineering, Auckland University of Technology, Auckland 1010, New Zealand; 4 Department of Electrical Engineering, College of Engineering, Taif University, P.O. Box 11099, Taif 21944, Saudi Arabia; 5 Department of Information Technology, College of Computer and Information Sciences, Princess Nourah bint Abdulrahman University, P.O. Box 84428, Riyadh 11671, Saudi Arabia

**Keywords:** low-profile, notch band antenna, reconfigurable, smart electronics, ultra-wideband

## Abstract

This study describes the design and implementation of a small printed ultra-wideband (UWB) antenna for smart electronic systems with on-demand adjustable notching properties. A contiguous sub-band between 3–4.1 GHz, 4.45–6.5 GHz, or for both bands concurrently, can be mitigated by the antenna. Numerous technologies and applications, including WiMAX, Wi-Fi, ISMA, WLAN, and sub-6 GHz, primarily utilize these band segments remitted by the UWB. The upper notch band is implemented by inserting an open-ended stub with the partial ground plane; the lower notch band functionality is obtained by etching a U-shaped slot from the radiating structure. The basic UWB mode is then changed to a UWB mode, with a single or dual notch band, using two diodes to achieve reconfigurability. The antenna has a physically compact size of 17 × 23 mm^2^ and a quasi-omnidirectional maximum gain of 4.9 dBi, along with a high efficiency of more than 80%, according to both simulation and measurement data. A significant bandwidth in the UWB region is also demonstrated by the proposed design, with a fractional bandwidth of 180% in relation to the 5.2 GHz center frequency. Regarding compactness, consistent gain, and programmable notch features, the proposed antenna outperforms the antennas described in the literature. In addition to these benefits, the antenna’s compact size makes it simple to incorporate into small electronic devices and enables producers to build many antennas without complications.

## 1. Introduction

The ultra-wideband (UWB) region is a frequency band spectrum well-known for its numerous advantages, including its low complexity, low cost, extremely low spectral power density, higher data resolution, very low interference, and extremely high data transmission rate. Since 2002, after the allocation by the Federal Communication Commission (FCC) of frequency band 3.1–10 GHz for UWB communication, it has gained considerable attention from both academia and researchers [1]. As a core component of communication systems, antenna design plays a critical role in these systems; therefore, numerous antennas dedicated to UWB communication have been proposed [2]. Although the antennas presented in [3,4,5] have several advantages, such as compact size, stable gain, and omnidirectional radiation patterns, their application is limited, as they do not provide any solution to avoid interference with the licensed and unlicensed bands existing within the UWB spectrum. Due to advancements in communication systems, several sub-bands have come into existence inside the UWB region, including the 5G sub-6-GHz band (3.7–4.2 GHz) and the X-band (7–8 GHz) [6,7]. The presence of these bands suggests the design of UWB antennas having notch bands to minimize the interference problems [8].

Although stop band filters are well known for stopping specific bands, their presence with antenna geometry introduces problems such as impedance matching, increased losses, and the overall size of the antenna [9,10]. Thus, researchers have adopted techniques such as etching slots in the patch, defected ground structure, and metamaterials to achieve notch band characteristics [6,7,8,9,10,11,12]. In [6], a monopole antenna is presented for UWB application, and a U-shaped slot is etched on the patch to notch the WLAN band; however, no solution is proposed to notch the other sub-bands. In [7,8,9,10], researchers adopted different shapes of slots to achieve dual-notch bands at the WLAN band and X-bands. The antenna presented in [7] showed a more compact size as compared to that in [8,9,10], along with ease of controlling the notch band independently, but no solution is proposed in [7,8,9,10] to switch the dual notch band to a single notch band, as per the system requirements. Another antenna for UWB applications, using a dual notch, is presented in [11], exhibiting a larger dimension. The researcher did not propose any solution to control the notches independently, due to complex geometry. A UWB antenna with hexa-notched bands is presented in [12]; the authors used open-ended stubs and meandered line slots to achieve notched bands. However, the presented work requires a special fabrication tool due to the complex structure, which results in high cost, thus limiting its applications for use in places where many antennas are used.

Although several notch band antennas are discussed, their application becomes limited due to the unavailability of switching notch bands. Modern devices require a UWB antenna which can notch a sub-band from the UWB region according to user demand [13]. This increase in demand can be fulfilled easily by designing a UWB antenna having reconfigurable notch bands. Thus, several reconfigurable notch antennas have been presented in the literature [13,14,15,16,17,18]. In [13], a single notched band reconfigurable MIMO antenna for UWB application is presented, and the antenna only exhibits a notch band when the diode is switched. On the other hand, a reconfigurable single notch band antenna is presented in [14]; it shows a switchable notched band at 5G sub-6-GHz band and a WLAN band using diodes. However, no methodology is presented to achieve a dual notch band simultaneously. Another interesting design is presented in [15], in which the authors used the varactors diode to achieve a switchable notch band. Besides having a larger dimension, it also lags in achieving a single or dual notch band mode. The antennas reported in [16,17] can achieve single-band or dual-band notching. However, the use of copper tape for measurements limits its use in electronic devices; therefore, no methodology is provided for fabrication in practical applications. A triple notch-band reconfigurable UWB antenna utilizing diodes, along with slots to achieve a triple, dual, and single notch band is presented in [18]. While they exhibit a compact size and notch-band reconfigurability, the antenna does not cover the complete UWB band.

A compact UWB antenna that can achieve multiple notches and single notches on demand, while covering the entire UWB spectrum, is still a problem considering the current research efforts. As a result, this paper describes the conception and construction of a small dual notch band UWB antenna. To create UWB, a typical monopole antenna is modified utilizing a patch and a truncated ground. Additionally, a WLAN band notch is achieved using a stub, while a 5G sub-6-GHz band notch is accomplished using an inverted U-shaped slot. Finally, notch reconfigurability is achieved using two PIN diodes. The remaining sections of the article are as follows: in Section 2, the anticipated antenna’s structure is described, along with a brief explanation of the methods used to develop the work that is being presented. In Section 3, findings from simulations and measurements are compared, and the proposed work is compared with the current state of the art. In the final portion of the paper, the discussion is finalized.

## 2. Antenna Designing

**Figure 1:**
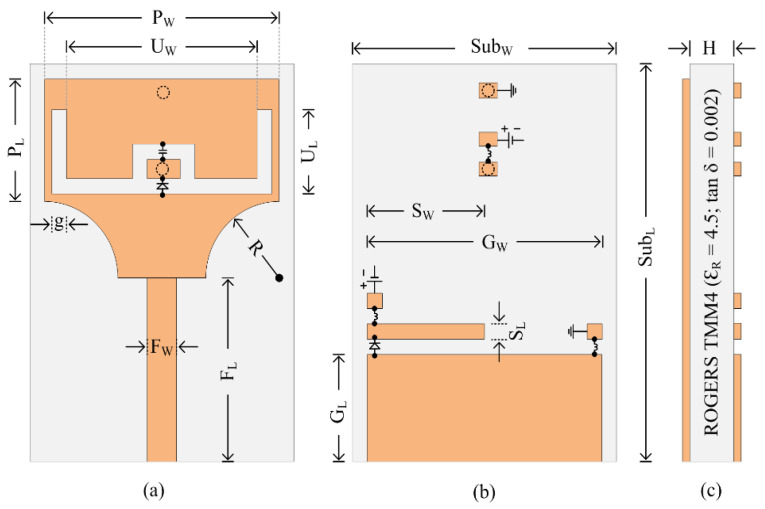
(**a**) Top-view; (**b**) bottom-view; (**c**) and side-view of the proposed reconfigurable notch band antenna.

The schematics of the proposed UWB antenna with the reconfigurable dual notch band are shown in Figure 1. The antenna geometry is engraved on the top side of ROGERS TMM4 substrate, with a tangent loss of 0.002 and an electric permittivity of 4.5. The substrate has an overall dimension of width (W) × length (L) × height (H). The bottom side of the substrate contains the partial ground plane, with a length of GL and a width of GW. The antenna radiator consists of a conventional rectangular patch having a length of PL and a width of PW, fed using a 50 ohm transmission line with the dimension of FL and FW. The lower end of the rectangular radiator was truncated using a cylinder of radius R and having a thickness equal to the copper cladding of the radiator, i.e., 0.035 mm. Afterwards, an inverted U-shaped slot having dimensions of UL and UW is utilized to achieve a notched band at the 5G sub-6-GHz band, while an L-shaped open stub with length SL and width SW is used to achieve the second notch band at the WLAN band. In the last step, two diodes are inserted in such a way that the antenna resonates with four different modes, i.e., a UWB antenna, a UWB antenna with notch band at 5G band, a UWB antenna with notch band at the WLAN band, and a UWB antenna with dual notch bands at the 5G and WLAN bands. The proposed antenna’s optimal dimensions are as follows: *Sub_W_* = 17 mm, *Sub_L_* = 23 mm, *H* = 1.524 mm, *P_W_* = 15 mm, *P_L_* = 7 mm, *G_L_* = 6.25 mm, *G_W_* = 15 mm, *R* = 5 mm, *F_L_* = 9.85 mm, *F_W_* = 1.6 mm, *U_L_* = 6.5 mm, *U_W_* = 13 mm, *S_W_* = 6.25 mm, *S_L_* = 1 mm, *g* = 0.5 mm.

A monopole-type antenna is used in this proposed paper due to the appealing physical features it presents. Indeed, monopole antennas are omnidirectional and are characterized by a simple structure, which facilitates their integration into miniature devices. Furthermore, monopole antennas are characterized by a stable radiation performance, with very wide bandwidths [19].

**Figure 2:**
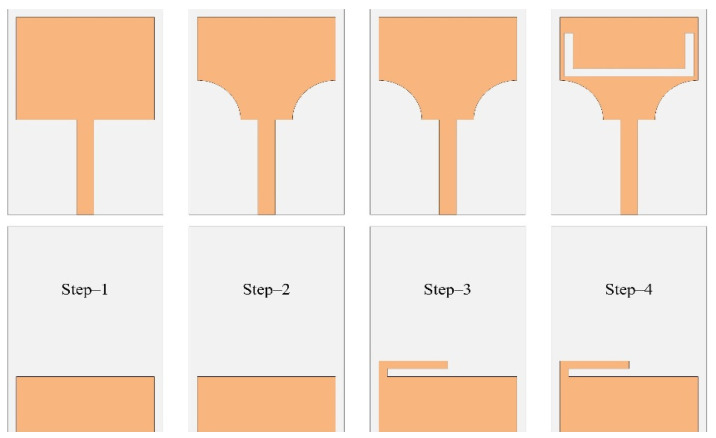
Design methodology steps of the proposed work.

Initially, a rectangular monopole antenna is designed, as shown in Figure 2, using the mathematical equations given in the literature. The reference antenna is excited by a 50 Ω microstrip transmission line to transfer the maximum power to the designed antenna.

The length of the basic monopole radiator is calculated using the formula given in [20].

(1) $$L=\frac{c}{2f_{c}\sqrt{\varepsilon_{reff}}}$$

where $L_{M}$ refers to the monopole radiator length, *c* is the velocity of light, $f_{c}$ presents the design frequency, while $\varepsilon_{reff}$ refers to the effective dielectric constant.

On the other hand, the resonance frequency of the antenna is calculated using the following equation:

(2) $$f_{c}=\frac{c}{\lambda_{g}\sqrt{\varepsilon_{eff}}}$$

where $\lambda_{g}$ is the guided wavelength, while $\varepsilon_{reff}$ refers to the effective dielectric constant, which is computed using the following equation:

(3) $$\varepsilon_{eff}=\frac{\left(\varepsilon_{r}+1\right)}{2}+\frac{\left(\varepsilon_{r}-1\right)}{2}\left[\frac{1}{\sqrt{1+12\frac{H}{W}}}\right]$$

where $\varepsilon_{r}$ denotes the dielectric constant, and *H* represents the substrate thickness.

To calculate the effective dielectric constant $\varepsilon_{reff}$, the value of the radiator width *W* must be calculated using the following equation [21]:

(4) $$W=\frac{c}{2f_{c}\sqrt{\frac{\left(\varepsilon_{r}+1\right)}{2}}}$$

Figure 2 shows the design evolution of the proposed antenna. A step-by-step analysis is performed for each design step to illustrate the method used to achieve the on-demand double notch band antenna and the effect of each notch on the performance of the proposed antenna. Different modifications of the antenna geometry were performed to obtain a maximum bandwidth covering the entire UWB region. Initially, a partial ground plane was used instead of a full ground plane to improve the bandwidth of the antenna towards lower frequencies [22]. The electrical length is then increased by truncating the bottom edges of the radiator to further increase the bandwidth of the antenna. Thereafter, a U-shaped slot is introduced into the radiator to mitigate the 5G-sub 6 GHz band, while a stub is inserted into the ground plane to notch the WLAN band from the UWB region. The physical dimensions of the antenna are obtained through a parametric study performed by simulations, then optimized using the genetic algorithm integrated into the 3D electromagnetic simulator HFSS.

### 2.1. UWB Antenna Design

**Figure 3:**
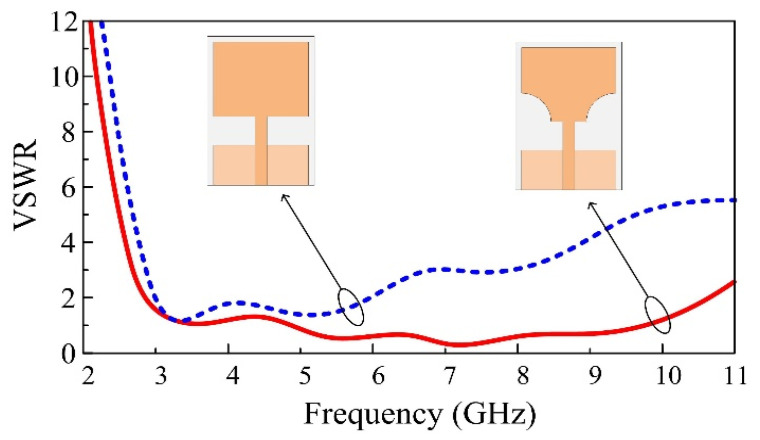
Comparison of a conventional rectangular monopole and a truncated corner UWB antenna.

Figure 3 presents the simulated VSWR of both full and truncated radiating elements. As can be noticed, the rectangular monopole antenna presents a VSWR value below 2 from 3.32 GHz to 5.95, i.e., 52% of FBW. The second step of the design consists of truncating two symmetrical cylinders of radius R from the bottom edges of the radiating element. The dimensions and the position of the truncated cylinders are determined through a parametric study to achieve optimal results in terms of bandwidth. In this case, the antenna shows perfect impedance matching along the entire UWB region, with a bandwidth ranging from 3 GHz to 10 GHz. The increase in the bandwidth is justified by the fact that the insertion of slots in the radiator increases the effective length of the antenna due to the increased fringing fields. This results in additional series inductance, which increases the bandwidth [23].

### 2.2. UWB Antenna with WLAN Notch Band

**Figure 4:**
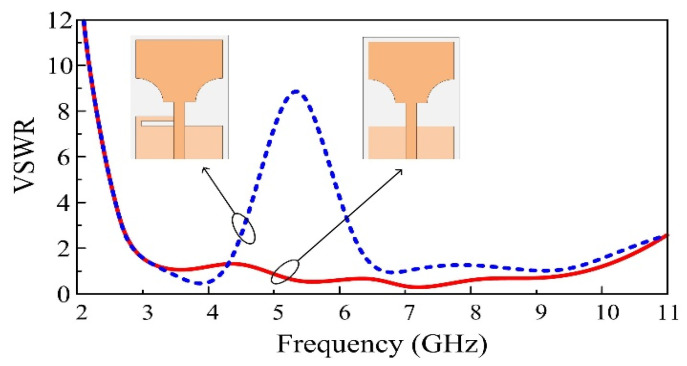
Comparison between UWB and WLAN notch band antennas.

The ultra-wideband (UWB) region is a frequency band region well-known for its numerous advantages, including its low complexity, low cost, extremely low spectral power density, high data resolution, very low interference, and extremely high data transmission rate [24]. However, one of the critical challenges of a band-notched antenna is the positioning of the notching structures within the antenna elements to mitigate the co-existing sub-bands [25]. In the present work, we introduced a stub in the antenna’s ground plane to block the WLAN sub-bands from interfering with the UWB region. The position and dimensions of the stub are chosen based on a parametric study carried out by simulations. As can be observed from Figure 4, the introduction of the stub in the antenna structure resulted in the attenuation of a sub-band from 4.6 GHz to 6.3 GHz, which corresponds to the WLAN bands, including 4.9–5.0 GHz (802.11j), 5 GHz (802.11a), 5.9 GHz (802.11p), as well as 85 channels of 6 GHz (802.11ax).

### 2.3. UWB Antenna with 5G Sub-6 GHz Notch Band

In the third step of antenna design, we introduced a U-shaped slot into the monopole radiator. In this case, the inserted notching structure allows the antenna to reject the 5G–6 GHz band, thus mitigating the interference of this sub-band with the UWB region. The dimensions of the notching slot are calculated using the following equation [26], while the slot’s location is optimized through a parametric study carried out by simulations.

(5) $$f_{notch}=\frac{c}{X_{0}\times L_{eff}\times \sqrt{\varepsilon_{eff}}}$$

where $f_{notch}$ refers to the central frequency of the U-shaped slot, *c* is the velocity of light in free space, $L_{eff}$ represents the effective length of the slot or stub, $\varepsilon_{eff}$ is the effective dielectric constant, while *X*_0_ is the multiplicative factor that represents the portion of the wavelength. The total effective length of the slot is *L_eff_* (U_L_ + U_W_ = 20.5 mm). When the value of *L_eff_* is put into Equation (5), the results will be equal to 3.59 GHz, which is very closed to the desire notch band of 3.5 GHz. The value of factor *X*_0_ in this case is 2, since the slot works at the half-wavelength at the desire frequency. On the other hand, the open-ended stub worked at a quarter-wavelength, so the value of factor *X*_0_ will be 4. Thus, after inputting the value of factor *X*_0_ and *L_eff_* (i.e., the length of stub = 6.25), the equation gives the value of 5.5 GHz, which is closed to the targeted value of the 5.2 GHz. The difference between the values is due to losses in the materials

**Figure 5:**
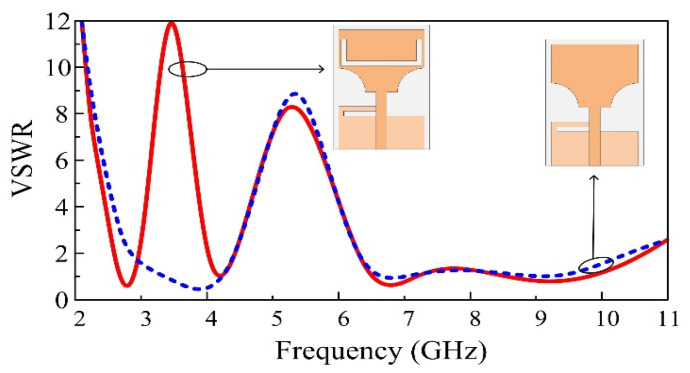
Comparison of single notch and dual notch UWB antennas.

The VSWR comparison between the antenna and the stub only and the antenna with both the stub and the U-shaped slots is illustrated in Figure 5. As can be observed, the introduction of the U-shaped slot in the monopole radiator has no impact on the overall performance of the antenna, except for providing a notched band that appears at the low frequencies. The antenna with the U-shaped slot shows an increase in VSWR around 3.5 GHz. As a result, we notice that the frequency band between 2.8 GHz and 3.83 GHz, which is particularly dedicated to 5G sub–6 GHz applications, is rejected from the UWB region.

### 2.4. UWB Antenna with Reconfigurable Dual Notch Band

As stated above, the introduction of band-notch features is indispensable to meet the UWB systems requirements. However, the ability to control the notch band is necessary. Introducing the notching structures must not interfere with the overall performance of the antenna. Therefore, independent controllable notched UWB antennas are required for modern wireless communication systems.

In the proposed antenna, PIN diodes are used to realize the reconfiguration function due to their compact size and high switching response. This can be explained by the fact that the electrical length of the radiator and ground plane change to allow for the appropriate current flow, depending on the state of the diode [9]. Therefore, to introduce the PIN diode in the proposed antenna structure, lumped resistance-inductor-capacitor (RLC) elements are used, and their values are listed in [27]. Moreover, a DC block 100 pF capacitor is used to change the path of the current flow [28]. In addition, vias and paddings are introduced to the antenna structure to achieve accurate results.

**Figure 6:**
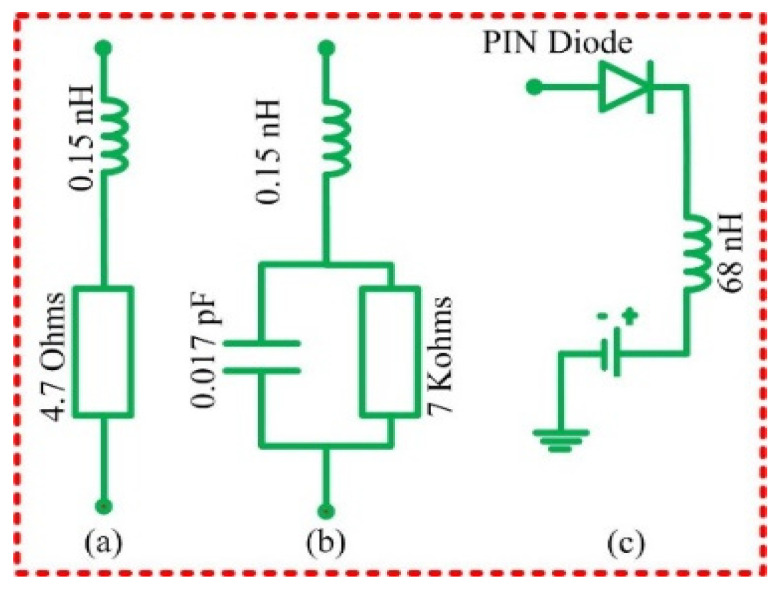
Equivalent circuit model of the (**a**) diode in the on-state, (**b**) diode in the off-state, and (**c**) biasing circuit to trigger the state of the diodes.

To implement electronic reconfigurability in the proposed design, we modeled the equivalent circuit of PIN diodes as depicted in Figure 6. A low-value resistor is connected in series with an inductance of 1.5 nH to allow for the current to flow in the “ON” state. Therefore, the electrical length of the radiator and the ground plane increases as the diode connects the conducting parts of the antenna. The reverse state, on the other hand, is illustrated in Figure 6b. In this case, a high-value resistor (7 KΩ) is mounted in parallel with a capacitor (0.017 pF); this parallel combination is then connected in series with an inductor, whose value is 0.15 nH. The high resistance and capacitance values prevent the flow of the current in the reverse bias, which explains the blocked state of the diode. Finally, Figure 6c shows the biasing circuit of the PIN diode. This circuit is composed of a DC source (which takes two values: 3 V in the forward direction and 0 V in the reverse direction), mounted in series with a resistor of 1K ohms to control the circulating current, then connected in series with an inductance of value 68 nH, whose purpose is to block the alternating current [29].

### 2.5. Optimization Algorithm

An evolutionary computational technique, called a genetic algorithm—distinguished by a high level of resilience and a comprehensive search of the solution space—is used to optimize the antenna described in this study. The design and optimization of the antenna geometry frequently use this kind of method. The system will choose the design parameters when the designer specifies the required performance of the antenna. The creation of the desired radiation pattern, lowering the side lobe levels of the radiation pattern, performing input impedance matching in a particular frequency or frequency range, or achieving the desired bandwidth for a specific application are all possible outcomes of designing the antenna using the genetic algorithm [30].

**Figure 7:**
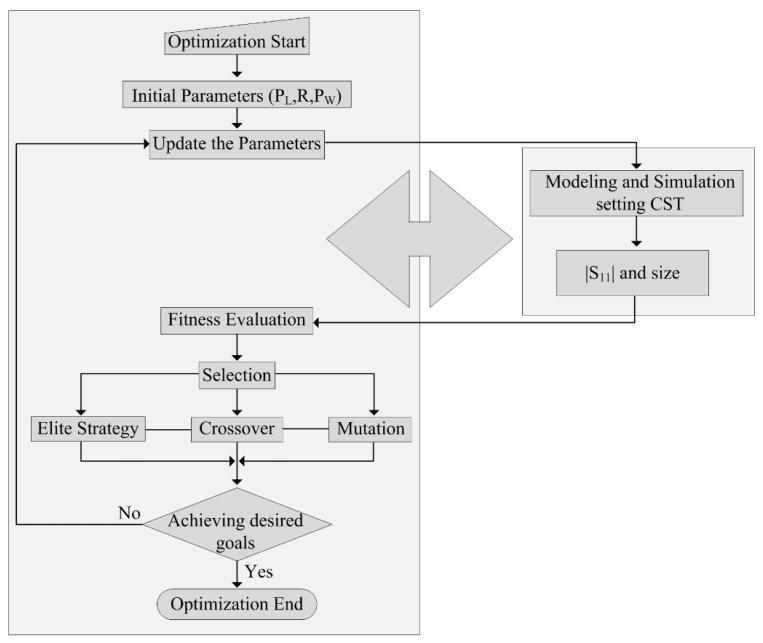
Flow chart illustrating the working principles of the optimization algorithm.

The basic idea behind the genetic algorithm is to select an initial solution of cells at random (individuals). The objective function and the fitness function are the two different types of functions used to assess each performance. The process of choosing the best chromosomes is started once the initial individuals are created. Next, the cells are coupled with one another to produce a new generation of chromosomes. This process is repeated over several generations until a particular requirement is satisfied [31]. A flow chart explaining the working methodology of the aforementioned algorithm is shown in Figure 7.

## 3. Results and Discussion

### 3.1. Simulation and Measurments Setup

The design, simulations, and optimizations of the proposed antenna are carried out using a 3D-electromagnetic solver HFSS (high-frequency structure simulator), mainly used to provide precise and consistent results for the most complex electromagnetic simulation challenges. The use of such a robust electromagnetic solver significantly reduces the number of tests and verifications required and avoids the construction of many prototypes, which considerably reduces the time and cost of product development. This software uses multiple methods, including the finite element method (FEM) and method of moments (MoM), which efficiently handle complex material and geometries.

The first step in using the HFSS electromagnetic simulator consists of choosing the solution type. In our case, the driven model solution is used to calculate the modal-based S-parameters of the monopole antenna. Next, a boundary condition is applied after designing a 3D model of the proposed monopole antenna to create and simplify the complexity of an electromagnetic model [32]. An SMA connector is then designed to minimize the connector’s impact on the overall performance of the antenna.

**Figure 8:**
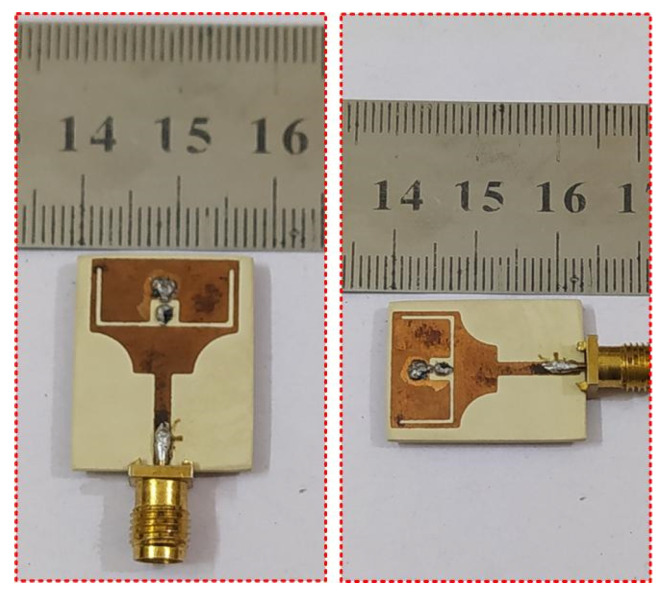
Fabricated prototype of the proposed antenna.

**Figure 9:**
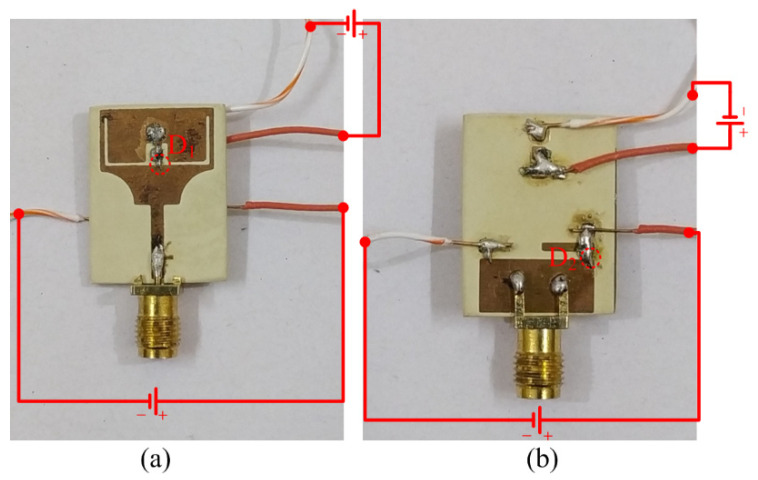
Fabricated prototype of the proposed antenna, along with biasing configuration; (**a**) top-view; (**b**) bottom view.

Figure 8 shows the fabricated prototype, without the DC supply wires. Figure 9a,b depicts the fabricated prototype with a complete biasing circuit and system, illustrating how the diodes are triggered to switch their state.

### 3.2. VSWR

**Figure 10:**
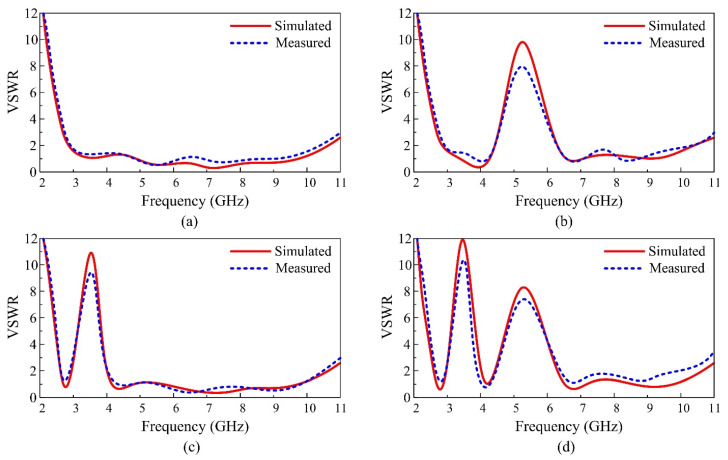
Comparison between simulated and measured VSWR for various switching states (**a**) case-10, (**b**) case-11, (**c**) case-00, and (**d**) case-01.

The proposed reconfigurable antenna’s simulated VSWR for the notched band and UWB scenarios is shown in Figure 10a–d. The antenna provides UWB when D1 is in the on-state, rendering the top notch inactive, and D2 is in the off-state, keeping the bottom stub electrically isolated from the ground plane. A 2:1 VSWR, regarded as moderately acceptable in low-powered applications in which power loss is more crucial, is shown in Figure 10a for case-10, demonstrating that the antenna covers a UWB bandwidth from 2.8 to 10.6 GHz.

As shown in Figure 10b, the antenna provides the UWB spectrum with a notched band that spans from 4.2 to 6.7 GHz for case-1, which corresponds to an active stub and an inactive slot. Notably, case-11 shields the UWB spectrums from the globally assigned band spectrum for Wi-Fi (4.9, 5, 5.9, and 6 GHz), Wi-Max (5.2 and 5.8 GHz), and ISM (5.8 GHz) applications. The antenna gives a notched band between 3 and 4.2 GHz for case-00, where the slot is active, but the stub is dormant, as shown in Figure 10c.

Finally, the presented antenna produces a dual notch band when both the stub and the slot are in operation with reference to diode case-01, as demonstrated in Figure 10d. The working band is between 2.7 and 10.2 GHz, while the lower notch band lies between 3–4.1 GHz, and the higher notch band is around 4.45 and 6.5 GHz. A substantial agreement between the anticipated and observed results is often seen for all switching states, validating the VSWR performance of the proposed antenna.

### 3.3. Radiation Pattern

**Figure 11:**
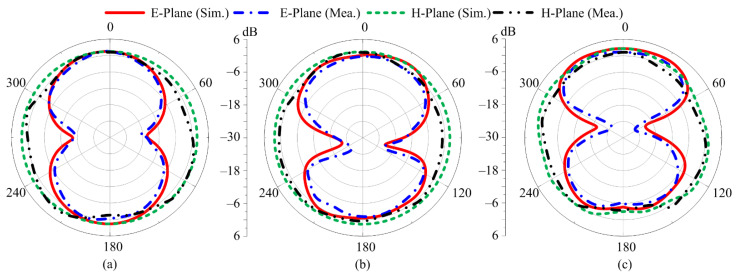
Comparison between simulated and measured radiation patterns for case-01 at the frequencies of (**a**) 2.8 GHz, (**b**) 4.2 GHz, and (**c**) 7 GHz.

The simulated gain radiation patterns at 2.8 GHz, 4.2 GHz, and 7 GHz are respectively shown in Figure 11a–c in the E-plane (θ = 00) and H-plane (θ = 900). The proposed dual notch band antenna reporting a far-field pattern reveals that at 2.8 GHz, the antenna exhibits a bidirectional radiation pattern in the E-plane and an omnidirectional radiation pattern in the H–plane. At 4.2 GHz and 7 GHz, a nearly omnidirectional pattern is observed in the H-plane, and a bidirectional pattern, with slight distortion, is observed in the E–plane, as depicted in Figure 11b,c.

### 3.4. Surface Current Distribution

**Figure 12:**
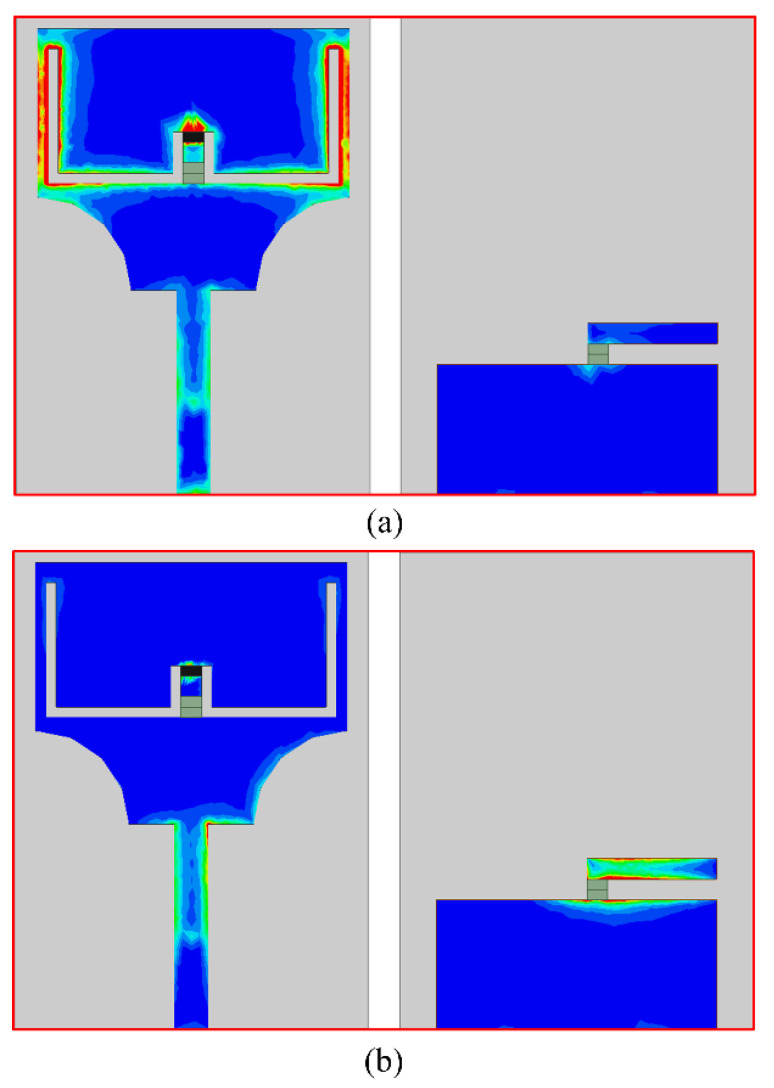
Surface current distribution of the proposed antenna for case-01 (**a**) 3.5 GHz; (**b**) 5.5 GHz.

One of the most important metrics utilized to evaluate the efficacy of the notch structures is the analysis of the current surface distribution, as illustrated in Figure 12a,b for case-01. The current is mostly focused on the etched slot and is evenly dispersed across the rest of the structure for the low notch band. In contrast, the current for the second notch frequency band is concentrated on the stub connected to a ground plane, proving that the lower and higher notch bands separately regulate the slot and stub.

### 3.5. Gain and Efficiency

**Figure 13:**
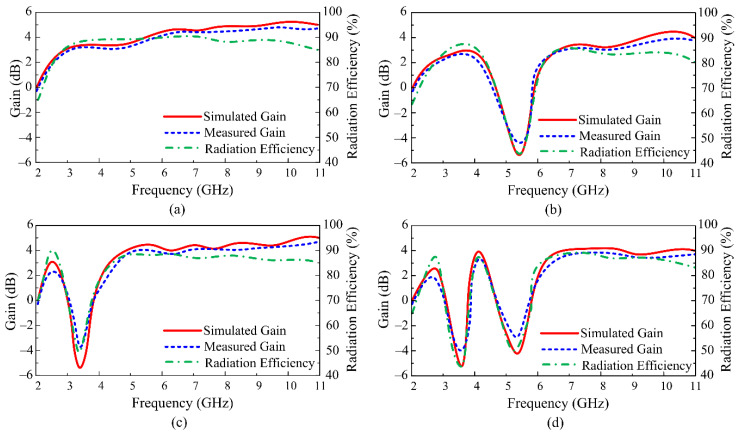
Comparison between the simulated and measured gain, along with simulated radiation efficiency, for various switching states (**a**) case-10, (**b**) case-11, (**c**) case-00, and (**d**) case-01.

For all feasible switching states of the diodes, the comparison between the simulated and observed gain findings is shown in Figure 13a–d. The antenna provides a gain of more than 2 dB for an operating bandwidth with a peak observed value of 4.9 dB near 9.6 GHz for the UWB scenario. Additionally, the radiation efficiency for the UWB operational mode is higher than 85%, as shown in Figure 13a. When the antenna operates with a single notch band, by switching the state of the diode in reference to cases-11 and -00, as depicted respectively in Figure 13b,c, the proposed antenna offers a gain of more than 2 dB in the resonating mode, whereas the gain tends to decrease in the notch band region, while attaining a minimum measured value of −4 dB. Similar results are shown for radiation efficiency, where the efficiency drops by up to 50% for the upper and lower notch in the notch band region, as shown in Figure 13b,c, respectively.

Lastly, for case-01, when the antenna offers both notch bands simultaneously, the gain of the presented antenna drops to −3.8 dB for the lower notch band, while the measured value shows that the gain is decreased up to −2.5 dB for the upper notch band, as depicted in Figure 13d. The radiation efficiency also shows a similar behavior, where the minimum value for the lower and upper notch band reaches up to 45% and 53%, respectively. The measured results offer a strong agreement with the simulated results in all switching states, showing the strong performance stability of the proposed reconfigurable notched band UWB antenna.

## 4. Comparison with the Related Works in the Literature

Table 1 presents the comparison of the state of the art with the proposed UWB antenna possessing a reconfigurable notch band. The proposed antenna offers a compact size, as compared to works in the literature, while covering the complete UWB spectrum (3.1–10.6), allocated globally. Moreover, the proposed work offers more modes of operation as compared to the work reported in [13,14,15]. Although the work reported in [16,17] offers a similar mode as compared to the antenna presented in this article, the usage of copper diodes limits their application for practical usage. Likewise, in [18], the antenna can mitigate three bands at a time, but does not provide the UWB operational mode, which also reduces its potential for applications requiring an antenna having both UWB and UWB with a notch band mode.

## 5. Conclusions

This paper focuses on the design and development of a compact printed UWB antenna having on-demand adjustable notching features for smart electronic systems. By cutting two circular slits from the bottom of the radiator, a rectangular-shaped monopole antenna with a partial ground plane is modified and optimized to provide UWB behavior. The next step is to attenuate the sub-bands of 3–4.1 GHz and 4.45–6.5 GHz from the UWB area, either individually or simultaneously, using first a U-shaped slot and subsequently, an open-ended stub. The antenna offers numerous advantages having a compact size of 17 × 23 mm^2^; the 2:1 VSWR value ranges from 2.8–10.6 GHz, showing a gain of more than 2 dB, and a radiation efficiency of more than 85%, while offering an omnidirectional radiation pattern. Moreover, the validation of the results and their comparison with the simulated results also show the performance stability of the proposed antenna. Furthermore, the on-demand reconfigurable notch band feature of the proposed work, along with the aforementioned feature, make it a strong candidate for smart electronics as compared to the work in the current literature.

## Figures and Tables

**Table 1:** Analysis of the proposed reconfigurable notched UWB antenna in comparison to cutting-edge antenna.

Ref. No.	Dimension (mm × mm)	Bandwidth (GHz)	Notch Band (GHz)	Reconfigurability Method	Operational Modes
[13]	39.8 × 23	1.8–12	4.8–6.2	PIN diode	UWB UWB with a single notch
[14]	42 × 32	2.2–12.5	3.1–4.5	PIN diode	UWB with a single notch
			3.6–8.2		
[15]	36 × 34	2.8–10.3	1.8–4.35	Varactor	UWB with two fixed and a single reconfigurable notch
[16]	32 × 24	2.7–12	4.4–5	Copper tape	UWB UWB with a single notch UWB with a dual notch
			7.7–9.2		
[17]	22 × 21	2.8–10.3	5.2–5.8	Copper tape	UWB UWB with a single notch UWB with a dual notch
			7.8–9		
[18]	25 × 21	3.1–10	3.2–4	PIN diodes	UWB with a single notch UWB with a dual notch UWB with a tri notch
			5.2–5.9		
			7.9–9		
Proposed work	17 × 23	2.8–10.6	3–4.1	PIN diodes	UWB UWB with a single notch UWB with a dual notch
			4.5–6.5		

## Data Availability

Not applicable.
